# Transition by head-on collision: mechanically mediated manoeuvres in cockroaches and small robots

**DOI:** 10.1098/rsif.2017.0664

**Published:** 2018-02-14

**Authors:** Kaushik Jayaram, Jean-Michel Mongeau, Anand Mohapatra, Paul Birkmeyer, Ronald S. Fearing, Robert J. Full

**Affiliations:** 1 Department of Integrative Biology, University of California, Berkeley, CA 94720, USA; 2 Biophysics Graduate Group, University of California, Berkeley, CA 94720, USA; 3 Department of Electrical Engineering and Computer Science, University of California, Berkeley, CA 94720, USA; 4 School of Engineering and Applied Sciences, Harvard University, Cambridge, MA 02138, USA; 5 Department of Mechanical and Nuclear Engineering, The Pennsylvania State University, University Park, PA 16802, USA

**Keywords:** climbing, mechanical control, robustness, biomechanics

## Abstract

Exceptional performance is often considered to be elegant and free of ‘errors’ or missteps. During the most extreme escape behaviours, neural control can approach or exceed its operating limits in response time and bandwidth. Here we show that small, rapid running cockroaches with robust exoskeletons select head-on collisions with obstacles to maintain the fastest escape speeds possible to transition up a vertical wall. Instead of avoidance, animals use their passive body shape and compliance to negotiate challenging environments. Cockroaches running at over 1 m or 50 body lengths per second transition from the floor to a vertical wall within 75 ms by using their head like an automobile bumper, mechanically mediating the manoeuvre. Inspired by the animal's behaviour, we demonstrate a passive, high-speed, mechanically mediated vertical transitions with a small, palm-sized legged robot. By creating a collision model for animal and human materials, we suggest a size dependence favouring mechanical mediation below 1 kg that we term the ‘Haldane limit’. Relying on the mechanical control offered by soft exoskeletons represents a paradigm shift for understanding the control of small animals and the next generation of running, climbing and flying robots where the use of the body can off-load the demand for rapid sensing and actuation.

## 1. Introduction

It is generally held that an animal's seemingly flawless performance to manoeuvre around obstacles stems from the extensive reliance on neural feedback from multimodal sensory systems, along with the actuators to execute the response. However, during rapid locomotion, the effectiveness of such neural feedback in response to perturbations is likely to be reduced due to decreased reaction times available for sensing, feedback and recovery, thereby increasing the chances of failure and the risks of sustaining damage from collisions. An alternative strategy for control of high-speed animal locomotion relies on mechanically mediated navigation and feedback of near instantaneous responses from viscoelastic mechanical structures arising from dynamic animal–environment interactions.

Instead of avoidance, animals can use their passive body shape and compliance to negotiate challenging environments. For instance, Li *et al*. [1] showed how fast running cockroaches head straight into multicomponent, three-dimensional terrain composed of grass-like, vertically compliant beams. The cockroaches' ‘terradynamically streamlined’ fusiform shape causes them to execute a novel roll manoeuvre—a form of natural parkour—facilitating rapid traversal of vertical gaps narrower than half their body width. Exploiting the terrain's properties can enhance traversability by assisting effective body reorientation via distributed mechanical feedback. Jayaram & Full [2] discovered that cockroaches can capitalize on their soft-bodied, shape-changing ability to traverse horizontal crevices smaller than a quarter of their height in less than a second by permitting the compression of their bodies' compliant exoskeletons in half.

Given the diversity in size of animal bodies, spanning over 10 orders of magnitude in mass [3], and its constituent materials ranging from soft to stiff, and brittle to tough [4], the dynamic responses from such mechanical structures, and, consequently, their effectiveness in mitigating the effect of obstacle collisions during locomotion must vary. Certainly, mechanically mediated strategies for negotiating obstacles must be size dependent. As observed by Haldane [5], ‘you can drop a mouse down a thousand-yard mine shaft; and, on arriving at the bottom, it gets a slight shock and walks away. A rat is killed, a man is broken and a horse splashes,’ pointing to the fact that the cost of collision damage increases with the size of the animal.

To escape from predators, cockroaches run at speeds approaching 1.5 m s^−1^ [6], climb up walls [7], race along ceilings [8] and then ingress into narrow crevices [2]. Aided by their low mass and moment of inertia, cockroaches can rapidly change direction by turning [9,10] or disappear rapidly by swinging under ledges [11]. During these high-speed behaviours, collisions with the ground and obstacles in their environment are frequent. We question whether these behavioural observations of collisions should be characterized as ‘missteps’, ‘failures’ [12] or ‘disasters’ [13], but rather be considered an effective strategy as part of the speed versus accuracy trade-offs proposed by models of escape [14]. Here, we explore the capability of cockroaches to rely on the viscoelastic properties of their exoskeleton to negotiate a transition from horizontal ground running to vertical wall climbing via mechanically mediated collisions.

We selected the American cockroach, *Periplaneta americana*, because of its ability to seamlessly transition between running and climbing. Since it tends to use high-speed manoeuvres to escape [15,16], there is a high probability of collisions with obstacles and opportunities for using mechanically mediated strategies. To elucidate the mechanism of rapid horizontal to vertical transitions, we elicited an escape response towards a high-contrast vertical wall. Following Haldane's predictions [5], we hypothesized that body collision resistance decreases with an increase in size. We developed a model relating bulk mechanical properties such as stiffness, damping and damping ratio to performance metrics such as kinetic energy, coefficient of restitution and percentage energy dissipation as a function of body size. Further, using elastic energy [4] and toughness [17] as measures critical for preventing bodily injury and thus robustness, we estimate the Haldane limit—maximum body size for dissipating energy upon collision without damage. Inspired by the mechanically mediated cockroach transition strategy, we modified our palm sized robot (DASH, Dynamic Autonomous Sprawled Hexapod [18]) manufactured using Smart Composite Microstructures (SCM, [19]) to perform rapid horizontal to vertical transitions by relying only on viscoelastic responses from its tuned body structures. The reliance on the body's mechanical mediation of obstacles represents a paradigm shift for understanding the control of small animals and the next generation of mesoscale and smaller running, climbing and flying robots.

## 2. Material and methods

### 2.1. Animals

We used 18 male cockroaches *P. americana* (Carolina Biological Supply, Burlington, NC, USA) with an average mass of 0.71 ± 0.13 g (mean ± s.d.). Prior to experimentation, cockroaches were kept in communal plastic containers at room temperature (22°C) on a 12 h : 12 h light dark cycle and provided water and food (fruit and dog chow) *ad libitum*.

### 2.2. Track and climbing surfaces

To demonstrate horizontal to vertical transitions, we constructed a horizontal acrylic track—100 cm long and 10 cm wide (electronic supplementary material, figure S1). The sidewalls of the track were coated with petroleum jelly to prevent the cockroach from climbing. The running surface was lined with paper for the standard condition to ensure adequate friction. A vertical wall made of hard posterboard (Royal Brites, US) 10 cm high was placed across the track to elicit a transition. Our preliminary experiments showed no effect of wall properties. The vertical wall had a black and white checkerboard design to provide a high contrast for visual detection.

### 2.3. Kinematics

We recorded videos of cockroaches running on a level surface, transitioning to a vertical posture, and climbing the wall using synchronized high-speed video cameras (AOS X-PRI, AOS Technologies, Switzerland) recording at 500 frames per second (fps) at a resolution of 1280 by 1024 pixels. One camera was positioned directly above the track, capturing the top view, and the other recorded the side view. Additionally, the track was evenly lit with minimal shadows using diffusers and two large high-power flood lamps (Lowel, Brooklyn, NY, USA) located on either ends of the track. We determined the kinematics of the transition from the captured videos using a motion tracking software package (Pro Analyst v. 6, Itronx Imaging Technologies, Westlake Village, CA, USA).

### 2.4. Animal experimental protocol

All experiments were performed at 28 ± 2°C (mean ± s.d.). Before starting any experiment, a total of four kinematic markers (small dots of white liquid paper, BIC Wite-out) were placed on the pronotum and the abdomen (one each on dorsal surface and the side at both positions) to aid in the motion tracking. The top (or dorsal) markers were used to calculate running velocity and yaw, whereas the side markers were used to estimate body pitch. To encourage the animals to run and climb up the wall, we evoked a stereotypical escape response by light stimulation of their cerci or by gently blowing using a gas duster (Dust-off Inc.). We accepted trials when the animal ran rapidly and transitioned successfully onto the vertical wall. We rejected trials where (i) the cockroaches stopped or climbed the side-wall within 25 cm of the vertical wall or during the transition, (ii) their body (excluding their legs) collided with the side-wall, or (iii) exhibited turns of more than 15° during the run or while transitioning.

Cockroaches with intact antenna, compound eyes and ocelli, running on a paper surface with wall preview distance (available track length) of about 55 cm, under ambient lighting conditions represented our standard or control condition (seven animals). To ensure that these particular conditions were not biasing the behaviour of the cockroaches, we varied lighting conditions, visual input, wall preview distance and type of running surface. From the additional pool of 11 animals, four were randomly selected for each of the following treatments: lighting, visual input, wall preview distance and running substrate. Experiments with blinded animals to test the effect of visual input were performed last and those animals were not returned to the pool for further experimentation. Since the same animals were run in the standard condition (ambient light) and varied condition (low light) just prior, we were able to use paired statistics for comparison (table 1).

**Table 1.** Data for the transition experiments performed under different conditions for the head-first and body-angled transitions. Head-first is the dominant strategy used by the cockroaches to climb onto the vertical wall. The transition times are similar for the two strategies whereas the running speeds before transition is higher when the animals perform the head-first transition. For transition times and running speeds, we show mean ± s.d.

control	condition	# trials [# animals]		transition times (ms)		running speeds (cm s^−1^)	
		head-first	body-angled	head-first	body-angled	head-first	body-angled
none	standard^a^	47 [7]	10 [6]	73 ± 29	75 ± 24	97 ± 14	79 ± 10
light	ambient	16 [4]	1 [1]	84 ± 40	68	94 ± 16	75
	*low*	13 [4]	6 [4]	68 ± 23	92 ± 29	99 ± 16	81 ± 1
visual input	normal	15 [4]	4 [3]	94 ± 24	69 ± 21	94 ± 12	84 ± 12
	*blind*	17 [4]	4 [3]	76 ± 13	97 ± 11	101 ± 12	96 ± 2
wall preview distance	55 cm	9 [4]	6 [3]	75 ± 18	69 ± 26	100 ± 11	91 ± 10
	*80 cm*	7 [3]	1 [1]	98 ± 34	72	105 ± 10	78
	*30 cm*	12 [4]	7 [4]	94 ± 17	65 ± 13	90 ± 10	88 ± 7
running surface	paper	11 [4]	5 [3]	102 ± 24	91 ± 34	99 ± 15	88 ± 15
	*sandpaper*	12 [4]	4 [2]	99 ± 31	81 ± 14	80 ± 12	102 ± 36
	*felt*	12 [4]	6 [4]	105 ± 27	113 ± 31	90 ± 23	85 ± 16

^a^Standard: ambient lighting, intact vision, 55 cm wall preview distance, paper as running surface.

Italic text represents test conditions different from the standard.

We tested two lighting conditions—ambient and dark. ‘Ambient’ lighting condition was about 21 000 lux bright. Since cockroaches prefer dark conditions in nature, we tested the animals under low-light conditions to remove possible biases induced by the brighter environment. The ‘low-light’ condition was the minimum lighting that enabled high-speed video capture at 500 fps (approx. 200 lux).

To allow the cockroach sufficient time to detect the wall and prepare for transition, we varied the wall preview distance. We chose 80 cm as the upper limit of the wall preview distance because the cockroaches either slowed down or stopped during the runs of longer lengths. Thirty centimetres was chosen as lower limit to allow for a steady-state run satisfying our operational definitions. A mean value of 55 cm was used as the standard.

To test of the role of visual sensors involved in the transition behaviour, we blinded cockroaches by covering their compound eyes and the ocelli with white nailpaint, taking care to avoid the head/scape joint [20]. To test the role of mechanosensory antennal contact we attempted to modify the antennae. Unfortunately, any modifications resulted in animals reluctant to run. Finally, we switched the running substrate from the default paper to felt, a softer material and 40-grit sandpaper, a hard and rough surface to test for the effect of substrates.

### 2.5. Robot experimental protocol

We simulated a head-on impact transition using DASH by running the robot into the vertical wall at maximum speed (≈80 cm s^−1^). A cone shaped, inclined extension (20 mm wide)—henceforth referred to as the ‘nose’—was added to the front of DASH to facilitate the robot orienting upward upon wall collision (see electronic supplementary material, figure S2). Cardboard laminates (4-ply Railroad board, Peacock Inc.) with flexible, polymer (Dura-lar, Grafix Inc.) joints were used to construct the nose using the Smart Composites Manufacturing (SCM) process, the same technique used to build the rest of the robot. We lined the running surface with cork to provide effective traction and used a preview distance of 55 cm. We video recorded the robot using the same protocol described for the animals.

### 2.6. Coefficient of restitution estimation

To perform controlled head-on impacts, we suspended freshly deceased cockroaches like a pendulum at their centre of mass using light music wire (5/1000″; 30 cm in length). The cockroach pendulum reached speeds of about 1 m s^−1^ before collision similar to head-first impact transitions. A heavy brass paperweight (1 lb) was used as the wall into which the animals collided. The entire process was filmed at 1000 Hz providing us the time resolution to measure the velocities before and after impact. The rationale for these experiments was twofold. First, this allowed us to obtain consistent measurements of coefficient of restitution. Second, the measured energy losses during collision could be attributed to the passive mechanical properties of the insect exoskeleton and not active muscular actions, since we used freshly deceased specimens.

### 2.7. Data analyses and statistics

We analysed the data using custom software (MatLab, Mathworks Inc.). We performed statistics on animal data with at least five trials per experimental condition using Minitab (Minitab Inc.). We used repeated measures analysis of variance (ANOVA) and Pearson's chi squared (*χ^2^*) tests for continuous and nominal variables respectively. A repeated measures design with a mixed model was used to determine the effect of condition. In our model, the condition (head-first/body angled as the case may be) was included as a fixed effect while the animal was included as random effect. The response was our performance metric (running velocity, transition time, etc.).

## 3. Results

### 3.1. Strategies for transition

**Figure 1.**
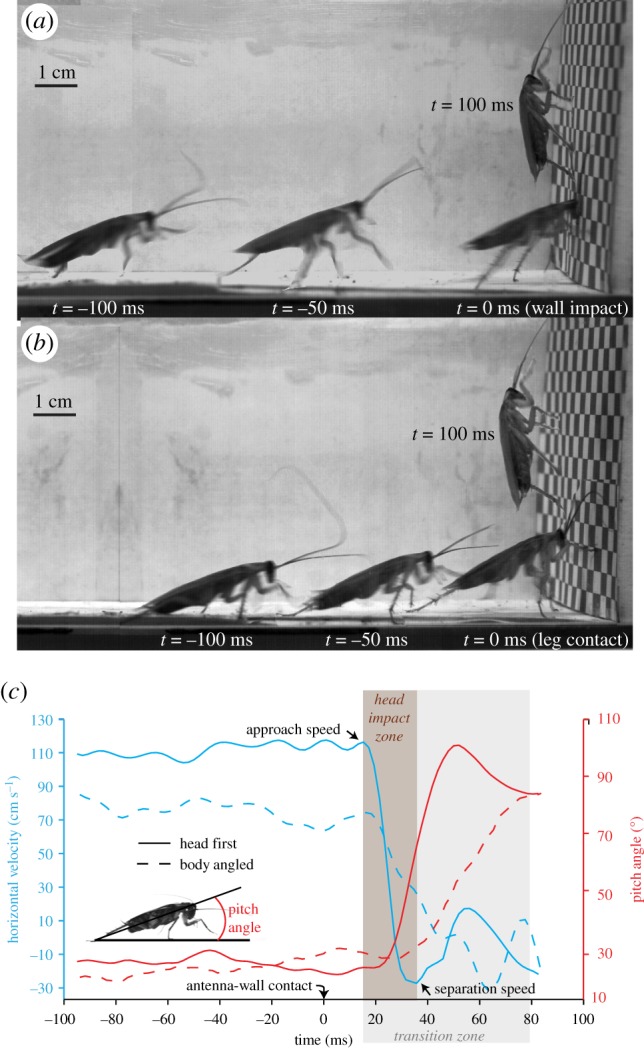
Time course of the two major high-speed transition strategies (approx. 75 ms). (*a*) Head-first impact strategy (table 1). Head-first impact is the primary (approx. 80%) transition strategy and often occurs at higher wall-approach speeds (≈1 m s^−1^). (*b*) Body-angled impact strategy. Cockroaches ran towards the wall with their body pitched head upwards and used their legs to decelerate and climb up the wall (table 1). (*c*) Horizontal running velocity and pitch angle during a typical head-first (solid line) and body-angled (dotted line) transition under the standard condition (intact vision, ambient light, 55 cm wall preview distance and paper as running surface). The transition zone is shown shaded in grey with the head impact zone further highlighted. Approach and separation speeds, which are the horizontal running velocities of the animal before and after head collision respectively, are also indicated.

Under the naked eye, cockroaches appeared to perform a smooth, ‘elegant’ transition onto the vertical wall. However, high-speed videography revealed two prominent transition strategies: head-first impact and body-angled impact. For the former strategy, cockroaches approached the wall at full speed, crashed head-on, before transitioning up the wall (electronic supplementary material, video S1). We refer to this behaviour as the head-first impact strategy (figure 1*a*). For the latter strategy, cockroaches ran towards the wall with their body pitched head upwards and used their legs to decelerate and climb up the wall (electronic supplementary material, video S2). We refer to this as the body-angled impact strategy (figure 1*b*). Under the standard condition (*n*= 18 animals, 107 trials), we observed that the head-first strategy represented by far the major portion of our trials (86/107, ≈80%). In the remaining cases, the animals employed the body-angled impact strategy to transition. Animals collided with the wall in over 90% of the total trials attempted. In extremely rare instances, cockroaches either jumped (4/330) or flew (1/330) towards the target. We found no effect of individuals on transition strategy for animals running under standard conditions (*n* = 18 animals, Pearson *χ^2^* test, *p* = 0.289).

To ensure that our standard conditions were not biasing behaviour, we varied the following experimental conditions: light, visual input, wall preview distance and running surface (table 1). We found no statistically significant differences (Pearson *χ^2^* test, *p* = 0.631) in the strategy across lighting conditions (low light or ambient), visual input (blinding or intact vision), wall preview distance (30, 55 or 80 cm) and running surface properties (sandpaper, paper or felt). Further, the animals used in the standard and above experimental groups showed no statistically significant differences in strategy (Pearson *χ^2^* test, *p* = 0.224). This allowed us to combine the datasets and reveal no effect of individuals (*n* = 18) on transition strategy (Pearson *χ^2^* test, *p* = 0.839). These results suggest that head-first impact is not an anomalous behaviour introduced by the experimental conditions.

### 3.2. Performance comparison for the transition strategies

To compare the transition performance for the two strategies under the standard condition, we measured transition time, the time from the first wall contact—excluding the antennae—to both hind-legs on the wall. Contrary to our expectations, the two strategies showed no statistically significant difference in the mean transition time (75 ± 28 ms; ANOVA, *p* = 0.635; table 1). This result indicates that head-first impacts do not pose a disadvantage to the animal in terms of transition times. It must also be noted that irrespective of the strategy used, the transition times are extremely brief (about 1–2 strides). As shown in figure 1*c*, irrespective of the transition strategy, the animals maintained steady horizontal velocities while approaching the wall. But during transition, the kinetic energy was rapidly dissipated and the horizontal velocity decreased to below zero within about 20–30 ms. We measured negative horizontal velocities which showed that some animals even bounced back after impacting the wall. Further, it is interesting to note that head-first transitions (65–148 cm s^−1^) occurred at significantly greater (ANOVA, *p* < 0.001; table 1) mean running speeds (averaged over at least 25 cm before first wall contact) compared to the body-angled transitions (51–92 cm s^−1^). Therefore, using the head-first impact strategy to transition is potentially advantageous to the cockroach as it allows the fastest running speed with no decrease in the transition time. Further, a typical transition was characterized by rapid changes in pitch angle following wall contact for both strategies (figure 1*c*). Prior to transition, we found no evidence of any characteristic changes in body pitch angle enabling body posture adjustment to facilitate a particular transition strategy. The lack of clear changes in horizontal velocity and body pitch angle as the animal approaches the wall suggests limited neural influences during horizontal-to-vertical transition.

**Figure 2.**
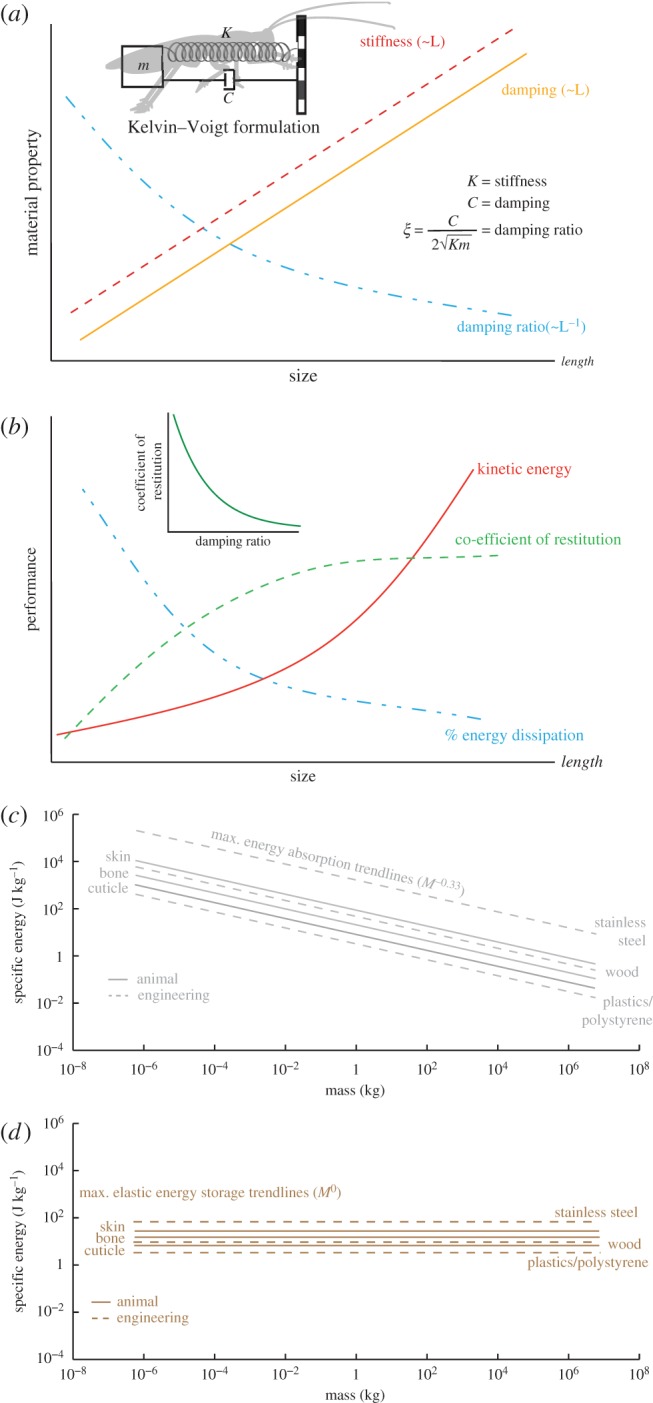
Scaling of mechanical behaviour to collisions. (*a*) Scaling of material properties—stiffness and damping and its consequence on damping ratio as predicted by an analytical model based on Kelvin–Voigt formulation. Stiffness and damping increase linearly with length, while damping ratio decreases with length. (*b*) Scaling of performance parameters—kinetic energy, coefficient of restitution (COR) and percentage energy absorption as predicted by an analytical model based on Kelvin–Voigt formulation. Inset indicates inverse relationship between COR and damping ratio [22]. Kinetic energy increases with size, as does COR indicating that percentage energy absorption decreases with size placing larger animals at greater danger of injury. (*c*) Log scaling of specific energy absorption determined using toughness versus body mass. Since toughness is constant for a material and independent of size, there is a linear decrease in specific energy with size. We show materials in animals (bone, skin and cuticle) and human technologies (wood, concrete, plastics). *M* represents body mass. (*d*) Log scaling of specific elastic energy storage determined using young's modulus and yield strength versus body mass. Since both these properties are constants for a material, elastic energy capacity is independent of body size. We show plots for materials in animals (bone, skin and cuticle) and human technologies (wood, concrete, plastics). *M* represents body mass.

To further characterize the head-first transition, we used the coefficient of restitution (COR) as our metric. COR is defined as the ratio of the velocity of separation to the velocity of approach [21] and is often used as a measure of kinetic energy loss (=1 − COR^2^) upon impact to describe the severity of collisions. For our case, we used the instantaneous running speed of the animal, one frame before and after head-contact as the approach and separation velocities respectively (figure 2). The mean COR for head-first impact transitions was 0.22, indicating that about 95% of the kinetic energy was dissipated by the cockroach exoskeleton. The independently measured COR using a cockroach pendulum was 0.26 ± 0.1 (2 animals, 14 trials), which is in close agreement with the experimental measurements.

### 3.3. Scaling of mechanical properties

Not all animals can use a head-first strategy to transition without severe injuries. Collision resistance, defined as the ability of a viscoelastic body to dissipate energy (as determined by the coefficient of restitution, COR) is size-dependent (figure 2). Assuming an animal's body to be composed of linear viscoelastic elements consistent with the Kelvin–Voigt model [17], we model the head-first impacts as a mass-spring-damper system whose dynamics are governed by the following second-order ordinary differential equation (equation (3.1)).3.1
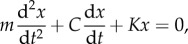
where *m*, *K*, *C* represent the effective body mass, spring constant (stiffness) and damping for deformation *x*, respectively. The above equation may be re-parametrized as follows (equation (3.2)) in terms of natural frequency (*ω*) and damping ratio (*ξ*).3.2
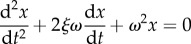
and3.3
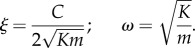


Damping ratio (equation (2.3)) is a dimensionless number indicative of how oscillations in a system decay after a disturbance. Several studies on impact pounding [22–24] have correlated damping ratio with the COR as an inverse relationship and it is therefore a measure of system's ability to dissipate energy. Using COR-damping ratio relationship [22], we estimate the damping ratio and natural frequency for a typical head-first transition (COR = 0.22) as *ξ* = 0.479 and *ω* = 377.96 rad s^−1^. We acknowledge that most of the above relationships have been derived for well-behaved engineering materials and additional detailed modelling likely will be required before adapting them to nonlinear biological materials. However, the inverse relationship between damping ratio and COR is expected to hold, and careful determination of the above relationship will aid in generating useful engineering design constraints as we discuss later.

Having established damping ratio as a proxy for COR, we can determine the dependence of damping ratio on mass, stiffness and damping, which scale with size (figure 2*a*,*b*). Assuming geometric scaling and homogeneous (isotropic) material composition, a structure, say cube of length, *l*, scaled ‘k’ times (*kl*) can be decomposed into ‘k^3^’ originally sized cubic units and arranged in ‘k’ layers in series, each composed of ‘k^2^’ such units. Therefore, using parallel and series laws, we obtain that stiffness and damping both increase with body length (*l*) and thus, the damping ratio decreases with length (*l*^−1^) (figure 2*a*). Therefore, a high damping ratio and consequently low COR value [22] places small animals at a definite advantage for impact mitigation because of their higher energy dissipation capabilities and lower kinetic energies (*l*^4^) relative to their larger counterparts (figure 2*b*).

To predict the scaling of specific energy absorption, another measure of body collision resistance, for a variety of materials used for construction of animals (bone, skin and cuticle) and human technologies (wood, concrete, plastics), we used elastic-plastic fracture toughness (*J* [4]). Since toughness (expressed in units of strain energy release per unit area [4,17]) is constant for a material and independent of size, we found that specific energy absorption (=(*JA*)/*M*), computed as the product of toughness (*J*) and cross-sectional area (*A*) per unit mass, decreased linearly with body length (*l*^−1^) (figure 2*c*). Likewise, we scaled specific elastic energy storage, yet another measure of collision resistance, using Young's modulus (*Y*), yield strength (*σ*) and material density (*ρ*). Since these properties are constants for a material, we found that the elastic energy storage capacity 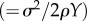 is independent of body size (figure 2*d*).

**Figure 3.**
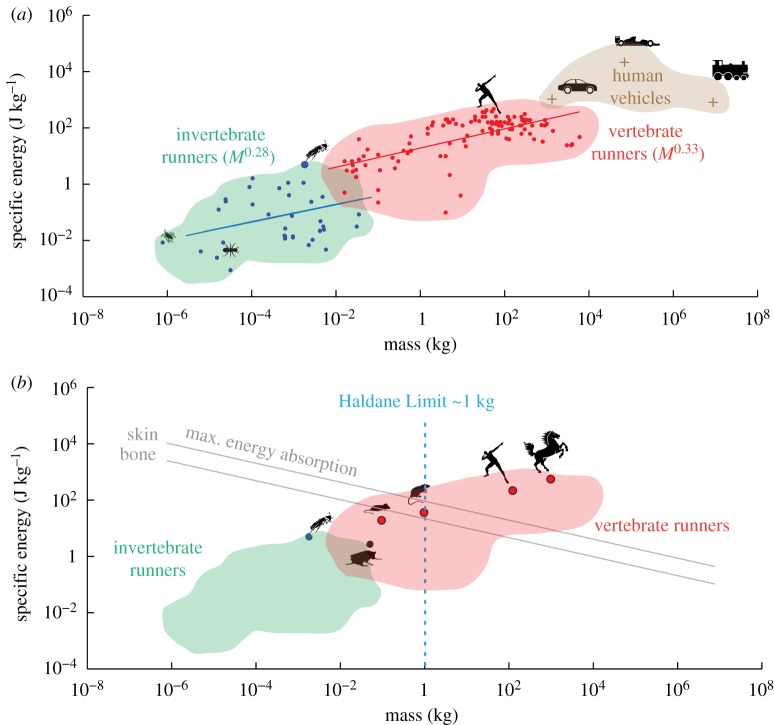
Scaling of robustness to collisions. (*a*) Log–log plot of the scaling of specific kinetic energy in terrestrial animals. Kinetic energy increases with mass exponentially and closely follows the trend predicted by inverted pendulum running (*M*^0.33^). Green cloud: invertebrate runners including cockroaches. Red cloud: vertebrate runners including humans. Tan cloud: human-engineered transportation (car, jet plane, train). (*b*) Haldane Limit Estimate. Plotting the data from figures (2*c* with 3*a*) shows that the curves intersect around 1 kg (blue line) quantifying the Haldane Limit. For sizes below 1 kg, animals might be able to absorb kinetic energy with their body materials, avoid injury during collisions and even select mechanical mediation of manoeuvres. However, for sizes to the right of the blue line, animals are less likely to completely dissipate their kinetic energy by material properties alone and would need to use mechanisms to either reduce speed if colliding into the environment or avoid collisions completely. Our model predictions match well with Haldane's observations about size dependence on energy dissipation and magnitude of injury upon collision. Our robot DASH (16 g) is well below the Haldane limit.

Further, we expect velocity (*v*) to scale as *M*^0.17^, where *M* is the mass, assuming dynamic similarity across animal sizes for an inverted pendulum locomotion template [25,26]. This prediction is in close agreement with studies on the scaling of maximum running speed of animals, estimated at *M*^0.17±0.04^ [27,28]. Using data from [27] and [28], the specific kinetic energy (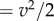) for an animal running at its top speed increased with body mass as *M*^0.33^ for vertebrates and as *M*^0.28^ for invertebrates respectively (figure 3*a*).

We then computed the cost of collision damage for an organism as the difference between its maximum possible specific kinetic energy at the time of collision and its maximum possible specific energy absorption given its constituent biological materials. To simplify the above calculation, we assumed animals were homogeneous cubes with uniform density of equal to that of water (1000 kg m^−3^) [29] that scaled geometrically with body size. The resulting plot (figure 3*b*) reveals that at the smallest sizes, energy absorption capacity dominates the kinetic energy, while at the largest sizes, kinetic energy overcomes absorption capacity. The intersection of the above trend lines yields the Haldane limit of about 1 kg. For animals larger than this critical body mass, it means that their entire kinetic energy cannot be fully dissipated without undergoing irreversible plastic deformation and such animals are therefore likely to incur significant body damage. Thus, this plot serves as indicator of the approximate size scales (below the Haldane limit) where mechanics and material properties can potentially influence obstacle avoidance behaviour. While the data presented here are mainly from cursorial animals, the performance-collision resistance trade-off is generic and may broadly hold across different modes of movement—flying, falling, parachuting and jumping. Numerous studies on medium to large sized vertebrates such as cats [30], dogs [31] and humans [32] are consistent with the limit. Figure 3*b* can be particularly useful for engineers to make initial designs, approximate choices about mass, material and geometry of robots, and lessen the burden on sensor based regulatory mechanisms to overcome perturbations or prevent collisions and damage.

### 3.4. Mechanically mediated transitions in a robot

**Figure 4.**
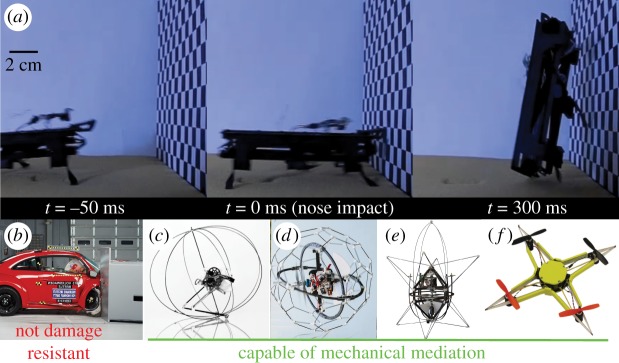
Mechanically mediated control in human technologies. (*a*) Dynamic Autonomous Sprawled Hexapod Robot (DASH) [19] performing a rapid head-first impact transition with no sensory input. Its robust construction enables it to perform high-speed manoeuvres without suffering damage while approaching the wall at over 80 cm s^−1^. (*b*) Volkswagen Beetle after incurring significant damages during a frontal impact crash test (Courtesy: Insurance Institute for Highway Safety, www.iihs.org). A typical coefficient of restitution for a front automobile bumper is ≈0.3 or 91% energy absorption. (*c*) Miniature (7 g) jumping robot [33] with self-recovery capabilities enabled by the robust exoskeletal cage. (*d*) Gimball robot with passive exoskeletal cage to use collisions for manoeuvring in cluttered environments [34]. (*e*) Airburr [35], an indoor flying robot designed specifically to withstand collision and self-manoeuvre using a shock-absorbing exoskeleton. (*f*) Insect inspired mechanically resilient multicopter [36] whose frame can undergo large deformations without permanent damage during collisions.

The robust exoskeletons of cockroaches provided inspiration for DASH (figure 4*a*). The robot without any kind of sensing collided with a wall at maximum speed (≈80 cm s^−1^) and performed a mechanically mediated transition (figure 4*a*, electronic supplementary material, video S3, COR ≈ 0.4), remaining undamaged. SCM technology [19] enabled DASH to not only be small (10 cm body length) and light (16 g), but also physically very robust allowing it to passively overcome obstacles and even sustain 8-story falls (over 28 m) without damage. Thus, we successfully demonstrated a passive, head-first impact transition using DASH as a physical model which supports the hypothesis that the cockroach head-first transition is a mechanically mediated manoeuvre. As next steps, we aim to incorporate substrate attachment mechanisms [37–39] into the feet of the robot in order to achieve climbing.

## 4. Discussion

Effective negotiation of the environment is most often characterized by smooth, nimble avoidance of obstacles. Yet, the American cockroach, *P. americana*, completed a high-speed horizontal to vertical transition within 75 ms (table 1) while suffering a head-on impact at maximum speed of 1.5 m s^−1^ into a vertical wall (figure 1*a*; electronic supplementary material, video S1). Even at half this speed, cockroaches have been observed to collide head-first into obstacles 10% of the time, despite being able to negotiate them using a single front limb movement without that limb ever touching the front of the obstacle [12,40]. Similar strategies during obstacle climbing have been observed at slower speeds in false death-head cockroaches, *Blaberus discoidalis*, and categorized as a head-butt [40] or as brute-force climbs where ‘the cockroaches pushed their head and body into an obstacle until that force resulted in its body pushing up and over the obstacle’ [41]. Baba *et al*. [12] found an increased frequency of collisions at higher ranges of speed (0.50 m s^−1^), along with the tendency to elevate the body. They state that, ‘It is tempting to suggest that these collisions represent failures to fully initiate a climb response despite the presence of the obstacle.’ Instead of a failure, a head-first impact transition may be potentially advantageous as it enables the animal to approach an obstacle or a vertical wall at highest possible speeds. Therefore, we contend that such collisions represent the animal's ability to use alternate mechanical mediation strategies rather than rely solely on neural feedback systems.

### 4.1. Selecting a mechanically mediated strategy for a maximal speed escape transition

The role of neural feedback in enabling escape behaviour has been studied extensively. In particular, cockroaches have been examined for their ability to follow walls using mechanosensory cues from their long antennae [20,42], avoid collisions during running by combining visual and antennal mechanosensory inputs [10], and even begin to escape from approaching predators using wind-receptive cerci in 60 ms [15,16]. These behaviours have been adopted as models for engineering control systems and sensors [43–45], and even inspired the development of crash avoidance systems for road vehicles [46]. Regardless of the multisensory arrays available, cockroaches in the present study predominantly crashed into the wall head-first to mediate the horizontal-to-vertical transition.

Although insufficient information exists to assess the field relevance of mechanical mediated transitions in this species, its origin in cave-like environments with walls/large rocks [47] is likely one reason this species predominantly adopts present day, human-made structures [48]. Changing the magnitude of sensory stimuli in our control experiments had no significant effect on the transition strategy, supporting the possibility that the behaviour is not unique to the laboratory. Specifically, varying light intensity and blinding the animals had no effect relative to controls (table 1). A weak effect of visual information agrees with earlier studies examining collision avoidance [12]. Similarly, we found no significant effect on speed or transition strategy when wall preview distance or running surfaces was varied (table 1). Previous studies [12] at slower escape speeds showed the importance of antennal mechanosensation in preventing collisions, since all manipulations altering the antennal system changed behaviour. Reducing antennal length, or severing the main antennal nerve without altering the length produced significantly increased collision frequency. These experiments found that nearly simultaneous contact with both antennae was required to make the cockroach stop and prevent a collision. Individuals in our experiment simply did not run with altered antennae. However, we suspect that antennal influences are minimal in our experiments because the typical time between the antenna contact to head impact was about 20 ms, which is of the same order as the neural conduction delays in antennae of *Periplaneta* [10] and faster than known antennae-touch escape responses (approx. 35–40 ms) (see [16] and its references). Therefore, under the extreme computational and bandwidth limitations of the nervous system, we could not find any evidence that cockroaches implemented sensor-based control. Instead, cockroaches relied on mechanically mediated control to negotiate the horizontal to vertical transition at maximum speed.

Running at maximal speed for escape is rare, especially for animals in the field, because the costs are considered to be too great [13]. Wynn *et al*. [13] state that ‘movement speed, even during extreme situations like escaping predation, should be based on a compromise between high speed, manoeuvrability, and motor control’. They ‘advocate that optimal—rather than maximal—performance capabilities underlie fitness defining behaviours such as escaping predators and capturing prey.’ and that slower speeds are selected to reduce the likelihood of ‘mistakes’ such as slipping, falling, and crashing. The reasons as to why the American cockroach does attain near maximal speed during a transition are likely complex. However, we contend that the present study removes crashing from the costs, and, instead, suggests that this mechanically mediated manoeuvre is a benefit allowing maximal speed running with minimal transition time and a low probability of injury.

### 4.2. The effect of size on collisions

Given the ubiquitous use of high-speed video, we now see that many more small animals undergo frequent collisions. Bees have been routinely observed to collide into walls at high-speed while attempting to enter hives [49]. Fruit flies experience head-on collisions and crash landings [50]. Coconut crabs habitually descend to the ground by jumping off trees [51]. Mosquitoes survive the high-speed impacts of raindrops [52], fire ants fall in their tunnels [53] and cockroaches crash land [54].

Haldane [5] attributed the different fates of a mouse, rat, man, and horse falling to relatively greater resistance to air in smaller animals owing to larger ratio of surface area to volume. Alternately, it can be argued that the terminal velocity [5,29] increases with body length (∼*l*^0.5^), and therefore, the speed of impact is higher for larger animals making them more susceptible to damage. Similarly, the maximum running speeds [26–29,55] of animals also increase with size (∼*l*^0.5^) resulting in higher kinetic energy (∼*l*^4^) in large animals leaving them vulnerable to head-on collisions [56]. Went [57] further argued that while infants trip and fall routinely and usually stay uninjured, adult humans are far more likely to end up with fractured bones because the momentum at ground contact upon tripping increases dramatically (∼*l*^5^). Using a Kelvin–Voigt model to represent an animal's body, we showed that the energy dissipation capability during head-on collision while running was size dependent leaving large animals at a further disadvantage. The maximum specific energy absorption (figure 3*b*) values calculated based on material toughness decreased with size (∼*l*^−1^) suggesting that except for invertebrates and a few small vertebrates, animals in general, are susceptible to permanent body deformation and bone fractures if involved in high-speed collisions. Therefore, mechanical properties favour small animals for survival during impacts [5,56] confirming one reason why cockroaches in the present study can use head-first impact transitions. Furthermore, this allows small animals to be less precise in controlling their behaviour, as the outcome in case of failure is not catastrophic compared to larger counterparts or traditional human-engineered technologies. Thus, animals with body sizes below the ‘Haldane Limit’, estimated to be about 1 kg, gain access to a variety of alternate, effective strategies that ensure successful performance.

### 4.3. Mechanically mediated transitions in robots

Biological studies have revealed that in dynamic, unpredictable environments, musculoskeletal structures [58] play a vital role in stabilizing locomotion [59] by managing any energetic deviations from steady state produced by perturbations from the environment [60–63]. Many of these principles have even been and continue to be adopted as models for engineering control systems [20,64]. Here, we have demonstrated a mechanically mediated transition at high speed using our hexapod robot DASH (figure 4*a*). The robot does not carry any sensors onboard and relies solely on the robust mechanical construction of its body elements to enable it to mitigate the impact and facilitate the transition. The role of such energy absorbing and deflecting body elements in control and manoeuvres of a robot is not limited to running, but has been successfully demonstrated during jumping (figure 4*c*, [33]) and flying (figure 4*d–f*, [34–36]). The analytical models developed in the impact studies [22–24] indicate an inverse relationship between damping ratio and COR, which means a high damping ratio correlates with low COR, i.e. a high-energy dissipation capability. This result highlights the importance of tuning the mechanical properties of the exoskeleton as it poses a trade-off between energy dissipation and possible energy redirection during mechanically mediated manoeuvres. In particular, such tuning would be critical to ensure successful performance during passive transition behaviours, especially in the case of robots inspired by the cockroach head-first transition.

Fortunately, advancements in meso-scale manufacturing technologies can now enable the production of robots in varying size scales with fine control over mechanical properties of individual body elements. Techniques such as Shape Deposition Manufacturing (SDM) [65], Smart Composite Microstructures (SCM) [19] and Printed Circuit Microelectromechanical Systems (PC-MEMS) [66] allow for precise machining and rapid prototyping of robots with dimensions in the centimetre scale [18,66,67]. Moreover, the above techniques offer the possibility of integration with the electronics, sensors and actuators during manufacturing [68], facilitating robots to robustly operate in real world environments, or allow them to be manufactured consistently and in high volume. In particular, flexure based millirobots, due to their inherent lightweight, low-loss joints and high-power densities, can easily be extremely dynamic and agile, making it possible to realize the amazing capabilities we see in nature's small animals. Therefore, they not only serve as ideal platforms for testing biological predictions, but also, can generate novel insights and testable hypotheses about biological systems.

### 4.4. Mechanically mediated control—a paradigm shift

By relying on the mechanics of the body to mediate manoeuvres rather than only careful sensor-based control makes animals and robots robust even under extreme conditions (figures 1 and 4). We see this as a paradigm shift in defining performance and contend that a successful performance must include a greater emphasis on morphological control and computation [69]. Although there remains contention as to if and what qualifies as actual computation, there is more of a consensus toward the notion of morphological control as described by Pfeifer & Bongard [70] where agents ‘off-load some neural processing into their morphology’. Hoffmann & Müller [71] point out that ‘the rich properties of ‘soft’ bodies (highly dimensional, dynamic, nonlinear, compliant and deformable) have been largely overlooked or deliberately suppressed by classical mechatronic designs, as they are largely incompatible with traditional control frameworks, where linear plants are preferred.’ Combining mechanical responses with neuromechanical feedback [58] involving multimodal sensory systems [72] leads to effective performance in biology. Incorporating the same in the design of robots can improve their overall robustness more significantly than regulatory mechanisms [73] added after the fact. Using this approach also overcomes the shortcomings resulting from limited response times (delays) during high-speed tasks in typical sensor based control systems in engineering. Furthermore, in a sensor based control system, the cost of recovery in such situations is significant [74,75] and often results in a failure at the intended task incurring irreversible damage to the system and environment. With the current trend of moving towards smaller, lighter and softer robotic platforms [76], nature tells us that we are likely to benefit from these more robust designs.

## Supplementary Material

FigS2_v2.pdf

## Supplementary Material

Movie_HighRes_Link.pdf

## Data accessibility

All relevant data are included in the manuscript. This article has no additional data.
