# Supplementary figures and images for: Transition by head-on collision: mechanically mediated manoeuvres in cockroaches and small robots
Source: J R Soc Interface. 2018 Feb 14;15(139):20170664. doi: 10.1098/rsif.2017.0664 (PMC5832722; doi:10.1098/rsif.2017.0664)

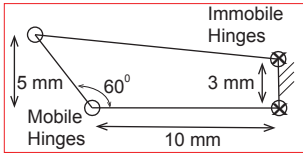

Rigidly connected  
to robot body

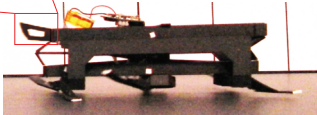

Supplement: FigS2_v2.pdf [file rsif20170664supp1.pdf]
