# Supplementary material for: Transition by head-on collision: mechanically mediated manoeuvres in cockroaches and small robots
Source: J R Soc Interface. 2018 Feb 14;15(139):20170664. doi: 10.1098/rsif.2017.0664 (PMC5832722; doi:10.1098/rsif.2017.0664)
Supplement: Movie_HighRes_Link.pdf [file rsif20170664supp2.pdf]

Transition by head-on collision: Mechanically mediated maneuvers in cockroaches and small robots

https://www.dropbox.com/sh/3dtfhc0ryafo038/AAB1zXE_dTrA6Xx04gh0xNJja?dl=0
